# Potential Antioxidative Activity of Homocysteine in Erythrocytes under Oxidative Stress

**DOI:** 10.3390/antiox12010202

**Published:** 2023-01-15

**Authors:** Mingxin Ye, Hui Li, Hongjun Luo, Yongyin Zhou, Wenhong Luo, Zhexuan Lin

**Affiliations:** Bio-Analytical Laboratory, Shantou University Medical College, Shantou 515000, China

**Keywords:** homocysteine, methionine, oxidative stress, antioxidant

## Abstract

Homocysteine is an amino acid containing a free sulfhydryl group, making it probably contribute to the antioxidative capacity in the body. We recently found that plasma total homocysteine (total-Hcy) concentration increased with time when whole blood samples were kept at room temperature. The present study was to elucidate how increased plasma total-Hcy is produced and explore the potential physiological role of homocysteine. Erythrocytes and leukocytes were separated and incubated in vitro; the amount of total-Hcy released by these two kinds of cells was then determined by HPLC-MS. The effects of homocysteine and methionine on reactive oxygen species (ROS) production, osmotic fragility, and methemoglobin formation in erythrocytes under oxidative stress were studied. The reducing activities of homocysteine and methionine were tested by ferryl hemoglobin (Hb) decay assay. As a result, it was discovered that erythrocytes metabolized methionine to homocysteine, which was then oxidized within the cells and released to the plasma. Homocysteine and its precursor methionine could significantly decrease Rosup-induced ROS production in erythrocytes and inhibit Rosup-induced erythrocyte’s osmotic fragility increase and methemoglobin formation. Homocysteine (but not methionine) was demonstrated to enhance ferryl Hb reduction. In conclusion, erythrocytes metabolize methionine to homocysteine, which contributes to the antioxidative capability under oxidative stress and might be a supplementary protective factor for erythrocytes against ROS damage.

## 1. Introduction

Homocysteine is a sulfhydryl group containing amino acid, which is produced within the body via the transmethylation of methionine. Briefly, methionine is converted into S-adenosylmethionine (SAM) in an ATP-dependent process. SAM then transfers its methyl group to various biomolecules, including DNA, proteins, and small molecules (such as phosphatidylethanolamine of the cell membrane) within cells. As such, the produced S-adenosylhomocysteine (SAH) is then hydrolyzed to homocysteine. Homocysteine can be metabolized to cysteine, converted back to methionine, or can form homocysteine-thiolactone [1]. Homocysteine is also released by cells to the extracellular fluid to prevent its intracellular accumulation [2]. It presents in its oxidized form (homocystine, homocysteine–cysteine, or protein-bound by disulfide bridges, about 98%) and reduced form (homocysteine, about 1%) in blood [3]. In this article, the reduced form of homocysteine is denoted as r-Hcy, the oxidized form of homocysteine is denoted as o-Hcy, and total-Hcy represents total homocysteine.

Vitamins B6, B12, and folic acid are required in the metabolism of homocysteine. As genetic background, for example, mutations involving 5,10-methylenetetrahydrofolate reductase or cystathionine β-Cysteine synthase affect plasma total-Hcy level. Aging has been reported to be positively correlated with elevated plasma total-Hcy levels [4,5]. A number of studies have shown that elevated plasma total-Hcy level is associated with Alzheimer’s disease, diabetes, chronic renal diseases, or atherosclerosis [6,7]. Therefore, the plasma total-Hcy level has been used as a metabolic disorder parameter in clinic, for which several methods have been developed to determine the concentration of total-Hcy in plasma [8,9]. A highly selective and sensitive high-performance liquid chromatography–mass spectrometry (HPLC-MS) method for the quantitative determination of plasma total-Hcy was developed in our previous study [10]. In the process when human blood samples were analyzed for total-Hcy in our laboratory, we found that plasma total-Hcy concentration increased gradually after whole blood samples were kept at room temperature for an extended time (i.e., more than 30 min). This phenomenon was previously reported by another study, indicating that plasma total-Hcy increased by 20% to 60% during storage of whole blood at room temperature for 4 to 24 h [11]. However, the cause of this phenomenon has never been clarified. 

Blood cells are the main components of blood and are still metabolically active and, therefore, are responsible for the metabolic changes of whole blood after being drawn from the body. Erythrocytes represent the most abundant cell type in blood, outnumbering the other circulating blood cells by several hundred times [12]. Therefore, we speculated that the increase of total-Hcy in blood samples after whole blood is kept for an extended time might originate from the metabolism of erythrocytes. Erythrocytes are highly susceptible to oxidative stress due to the high concentration of oxygen within the cells and other oxidants from the bloodstream [13]. However, erythrocytes possess enzymes (such as superoxide dismutase and catalase) and non-enzymatic antioxidative systems, which are able to minimize the oxidative damaging effect. Among them, sulfhydryl group (SH)-containing compounds, including reduced glutathione (GSH), act as intracellular redox buffers. Similar to GSH, homocysteine also contains a free sulfhydryl group (SH), making it very possibly an intracellular antioxidant. Although homocysteine-induced oxidative stress has been suggested in the cardiovascular system [14,15,16,17], researchers have been questioning these viewpoints [18,19].

Based on these considerations, the present study aimed to elucidate the main source of increased plasma total-Hcy during storage of whole blood at room temperature and to explore homocysteine’s potential antioxidative role in erythrocytes.

## 2. Materials and Methods

### 2.1. Reagents and Chemicals

DL-homocysteine, L-methionine, trichloroacetic acid (TCA), 2′,7′-dichlorofluorescein diacetate (DCFH-DA) were purchased from Sigma-Aldrich (St. Louis, MO, USA). DL-homocysteine-3,3,4,4-d4 (homocysteine-d4) was obtained from CDN Isotopes Inc. (Quebec, Canada). L-homocysteine was from Bide Pharmatech Ltd. (Shanghai, China). Hydrogen peroxide (30%), LC-MS grade acetonitrile, and methanol were from Merck (Germany). Catalase was purchased from Worthington Biochemical Corporation (NJ, USA). 2-chloroquinoline, trimethyloxonium tetrafluoroborate, and formic acid were purchased from Aladdin (Shanghai, China). Tris-(2-carboxyethyl) phosphine hydrochloride (TCEP) was purchased from Energy Chemical (Shanghai, China). Glutathione was obtained from Generay Biotech Co. Ltd. (Shanghai, China), and 2-chloro-1-methylquinolinium tetrafluoroborate (CMQT) was synthesized according to the literature [20]. Rosup (a chemical mixture including 4-Butylhydroperoxide, 50 mg/mL in DMSO, catalog S0033S-2) was purchased from Beyotime Biotechnology (Shanghai, China). The Methemoglobin Assay Kit was purchased from Nanjing Jiancheng Bioengineering Institute (Nanjing, China). The bovine hemoglobin and Ficoll-Paque solution were from Beijing Solarbio Science and Technology Co. Ltd. (Beijing, China).

### 2.2. Blood Sampling

Human blood was obtained upon informed consent from healthy volunteers (age 23~26 years) in 2 mL Blood Collection Tubes (Jinxing Ltd., Hubei, China) with the addition of 2.4 mg EDTA-K_2_. Informed consent was acquired according to our institutional guidelines and the Declaration of Helsinki. The study protocol and consent forms were approved by the Ethics Committee of Shantou University Medical College (No. SUMC-2020-22).

### 2.3. Determination of Plasma Total-Hcy after Whole Blood Kept at Room Temperature for Different Durations

The main purpose of this experiment was to confirm our previous finding that total-Hcy would increase after the whole blood was drawn from the body and kept at room temperature for an extended time. Whole peripheral blood (6 mL per volunteer) from 6 volunteers (three males and three females with ages between 23 and 26 years) was collected in tubes containing the anticoagulant, and each of them was divided into 12 tubes (0.5 mL per tube) immediately and kept at room temperature for 0 h, 0.5 h, 1 h, 2 h, 4 h, and 8 h. Samples from each time point included 12 tubes (2 from each individual) of blood. Plasma was then collected after centrifugation at 1000× *g* for 10 min and stored at −80 ℃ for determination of total-Hcy concentration.

### 2.4. The Production of Homocysteine from Blood Cells

The purpose of this experiment is to elucidate the main source of increased plasma total-Hcy during the storage of whole blood at room temperature. Erythrocytes and leukocytes were separated by a density gradient centrifugation method using Ficoll-Paque solution [21] and incubated in vitro. Briefly, peripheral blood (5 mL) was collected from a volunteer and gently layered on top of the Ficoll-Paque solution. After centrifugation at 1000× *g* at 4 °C for 30 min, the buffy coat (mix of leukocytes) in the interphase and Ficoll-Paque solution and the erythrocytes at the bottom were aspirated and washed three times with 0.01 M G-PBS (138 mM NaCl, 2.7 mM KCl, 8 mM Na_2_HPO_4_, 1.5 mM KH_2_PO_4_, 5 mM glucose, pH 7.4). Then, erythrocytes (2.4 × 10^9^ cells/mL) and leukocytes (2.9 × 10^7^ cells/mL) were kept in serum-free culture medium (RPMI-1640) in an incubator with 5% CO_2_ at 37 °C for 0 h or 3 h. The culture supernatant was then collected after centrifugation at 800× *g* for 10 min and stored at −80 °C for determination of total-Hcy. The results of total-Hcy were presented as nmol/10^6^ cells.

To determine the extracellular and intracellular concentrations of r-Hcy and o-Hcy, erythrocytes were freshly isolated from the peripheral blood (5 mL) of a volunteer by the density gradient centrifugation method as above. The erythrocytes were resuspended in plasma from the same volunteer at a density of 3 × 10^9^ cells/mL and then incubated at room temperature for 3 h. After incubation and centrifugation at 800× *g* for 10 min, the supernatant and erythrocytes were collected and stored at −80 °C for determination of total-Hcy and r-Hcy, from which the level of o-Hcy was calculated by subtraction of r-Hcy from total-Hcy.

### 2.5. The Production of Total-Hcy from Erythrocytes after Incubation with Methionine

The freshly isolated erythrocytes (3 × 10^9^ cells/mL) from peripheral blood (5 mL) of a volunteer were incubated with different concentrations of methionine (0, 0.01, 0.1, 1, or 10 mM in G-PBS) for 3 h in an incubator with 5% CO_2_ at 37 ℃. After incubation, the culture supernatant was collected after centrifugation at 800× *g* for 10 min and stored at −80 ℃ for determination of total-Hcy contents.

### 2.6. The Effects of r-Hcy and Methionine on Reactive Oxygen Species (ROS) Production in Erythrocytes

Rosup was widely used to induce oxidative stress in cell studies [22,23]. It was employed in the following experiments to produce ROS in erythrocytes. The freshly isolated erythrocytes from the peripheral blood of 3 volunteers (2 mL per volunteer) were diluted by G-PBS to a density of 5 × 10^6^ cells/mL and then incubated with 50 μM DCFH-DA for 30 min at 37 °C in the dark. After being rinsed twice with G-PBS, the erythrocytes were then treated with different concentrations of methionine (0.1, 0.2, 1, 5 mM in G-PBS) or r-Hcy (12.5, 25, 50, 100 μM in G-PBS) for 0.5 h, 3 h, and 6 h in the presence or absence of Rosup (final concentration of 50 μg/mL in G-PBS). Then, the ROS level was detected using a fluorescence microplate reader (Tecan Infinite^®^200 Pro, Salzburg, Austria). The excitation and emission wavelengths were set at 488 nm and 525 nm, respectively.

### 2.7. Measurement of Erythrocyte Osmotic Fragility

Erythrocyte osmotic fragility is an indirect method of assessing oxidative stress that can impair membrane stability [24,25]. The freshly isolated erythrocytes from the peripheral blood of volunteers (2 mL per volunteer) were suspended in G-PBS at a density of 6 × 10^8^ cells/mL and treated with different concentrations of methionine (0.1, 0.2, 0.5, 1, 2 mM in G-PBS) or r-Hcy (0.05, 0.1, 0.2, 0.4, 0.8 mM in G-PBS) in the presence of Rosup (50 μg/mL) for 6 h at 37 °C. Then osmotic fragility measurements were performed by using a series of sodium chloride solutions (NaCl, 0.2%–0.9%) [26]. Aliquots (50 μL) of erythrocyte suspension were added to a series of osmotic fragility solutions (450 μL), and the cell suspension was allowed to equilibrate at 37 ℃ for another 30 min. After centrifugation at 800× *g* for 10 min, the supernatant was determined for absorbance (A) at 540 nm. Erythrocytes treated with deionized water were regarded as 100% hemolysis. The rate of hemolysis was calculated using the following formula:

$$\%\;\mathrm{Hemolysis}=(A_{\mathrm{Sample}}-A_{\mathrm{Blank}})/(A_{100\%\;\mathrm{hemolysis}}-A_{\mathrm{Blank}})\;\times \;100\%.$$

The erythrocyte osmotic fragility curves were then constructed, and the NaCl concentrations that cause 50% hemolysis were determined.

### 2.8. The Effects of r-Hcy or Methionine on Ferryl Hb Decay

Hemoglobin (Hb) oxidation occurs with the formation of different redox states of Hb, including ferric Hb (Fe^3+^) and ferryl Hb (Fe^4+^). Ferryl Hb (Fe^4+^) can be reduced to form ferric Hb (Fe^3+^), which can be respectively measured by absorbance at 425 nm and 405 nm [27,28]. Briefly, 20 μM bovine Hb was pretreated with hydrogen peroxide (H_2_O_2_, 1 mM) for 15 min in phosphate-buffered saline (PBS, 0.1 M, pH 7.4) to form ferryl Hb (Fe^4+^), to which catalase (50 μg/mL) was added to remove the remaining H_2_O_2_. The optical changes from 350 nm to 700 nm were monitored, and the absorbance values of 405 nm and 425 nm were used to calculate the decay of ferryl Hb (Fe^4+^) after the addition of various concentrations of r-Hcy (0.025, 0.05, 0.1, 0.2, 0.4, 0.8 mM), or methionine (0.1, 0.2, 0.5, 1, 2 mM). Deionized water was used as blank control.

### 2.9. Measurement of Methemoglobin (MetHb) in Erythrocytes

The freshly isolated erythrocytes from the peripheral blood of 3 volunteers (2 mL per volunteer) were suspended in G-PBS. Then, erythrocytes (6 × 10^8^ cells/mL) were incubated with different concentrations of methionine (0.5, 1, 2 mM in G-PBS) or r-Hcy (0.05, 0.1, 0.2, 0.4, 0.8 mM in G-PBS) in the presence of Rosup (50 μg/mL) for 1.5 h, 3 h, 6 h, and 9 h. The cultures were then assayed following the guideline of the MetHb Assay Kit. The absorbance of each sample at 630 nm represented the level of erythrocytes MetHb [29].

### 2.10. The Contents of Total-Hcy and Glutathione (GSH) in Rosup-Treated Erythrocytes

This experiment investigated whether treatment with methionine could restore the intracellular reduced form of GSH level under oxidative stress. The freshly isolated erythrocytes from the peripheral blood of 3 volunteers (2 mL per volunteer) were treated with Rosup (50 μg/mL) in the presence or absence of methionine (2 mM) for 3 h and 6 h. Then, the supernatant and erythrocytes were harvested after centrifugation at 800× *g* for 10 min and stored at −80 °C for total-Hcy and GSH determination.

### 2.11. HPLC-MS Determination of Total-Hcy and r-Hcy Concentrations

Total-Hcy concentration was determined using a Shimadzu LC30AD HPLC system (Shimadzu Corporation, Kyoto, Japan) coupled with Triple Quad 6500 System (Sciex, Framingham, MA, USA), as we previously described [10]. Briefly, 200 μL of plasma, culture supernatant, or cell lysate was added with 10 μL homocysteine-d4 solution (200 μM for plasma and 20 μM for culture supernatant or cell lysate) as internal calibration standard, and 20 μL reducing reagent TCEP (0.1 M). The mixtures were then maintained at room temperature for 10 min. Next, 200 μL PBS (0.2 M, pH 5.0) and 20 μL derivatization reagent CMQT (0.1 M) were added, vortexed, and kept at room temperature for another 5 min. Then, 110 µL 20% TCA was added to precipitate proteins. After centrifugation at 12,000× *g* for 10 min, 400 µL supernatant was subjected to a solid-phase extraction process (SPE, ProElut™ C18, size 100 mg; Dikma Technologies, Lake Forest, CA, USA). Each SPE column was activated by 2 mL of methanol and 3 mL of 50 mM TCA before use. After sample loading, it was washed by 500 μL TCA (50 mM), 1 mL methanol-TCA (50 mM, 7:93, *v/v*), and 2 mL methanol-TCA (50 mM, 5:95, *v/v*) successively. Finally, 200 µL acetonitrile–0.1% formic acid (3:7, *v/v*) was used to elute the derivatives, which were then analyzed by HPLC–MS/MS. For r-Hcy determination, 20 μL deionized water replaced TCEP during sample processing. The collected erythrocytes in 500 μL PBS (0.01 M, pH 7.4) were homogenized using an Ultra-sonic Homogenizer (Sonics, Newtown, CT, USA) to prepare the cell lysate.

HPLC separation was performed on an Agilent RRHD Eclipse Plus C18 column (2.1 × 50 mm, 1.8 μm) with column temperature set at 40 °C. The mobile phase was methanol—0.05% formic acid (15:85, *v/v*), and the injection volume was 2 μL. Positive ion mode (ESI+) was used for MS detection, and Multiple Reaction Monitoring (MRM) mode with the precursor ions and the fragment ions of homocysteine-CMQT derivative (277/176, *m/z*) and homocysteine-d4-CMQT derivative (281/176, *m/z*) were used for quantification.

### 2.12. HPLC Determination of GSH

Cellular GSH level was determined using the HPLC system as previously described [9,30]. Briefly, a 200 µL incubation supernatant or cell lysate was kept at room temperature for 10 min after the addition of 20 µL 0.1 M TCEP or deionized water. Next, 200 μL PBS (0.2 M, pH 5.0) and 20 μL CMQT (0.1 M) were added, vortexed, and kept at room temperature for 5 min. Then, 110 µL 20% TCA was added, vortexed, and centrifuged at 12,000× *g* for 10 min. The supernatant was then analyzed by HPLC (Agilent 1100, Santa Clara, CA, USA) with an Agilent Zorbax SB-C18 column (4.6 × 150 mm, 5 μm) at 30 °C. The mobile phase was 50 mM TCA (pH 1.65 adjusted with 50 mM lithium hydroxide)–Acetonitrile (85:15, *v/v*) at a flow rate of 1 mL/min and the injection volume was 20 μL. The wavelength of the UV detector was set at 350 nm.

### 2.13. Statistical Analysis

Data are presented as the means ± standard deviation (SD). Statistical analysis was performed with GraphPad Prism software (version 8.00 for Windows; San Diego, CA, USA). The normality of data was evaluated by the Kolmogorov–Smirnov test. Analysis of Variance (ANOVA) with post-hoc Tukey’s multiple comparisons test was used for comparison of means between groups. *p* < 0.05 was considered statistically significant.

## 3. Results

### 3.1. The Plasma Total-Hcy Increased after Whole Blood Samples Kept at Room Temperature for Different Durations

**Figure 1:**
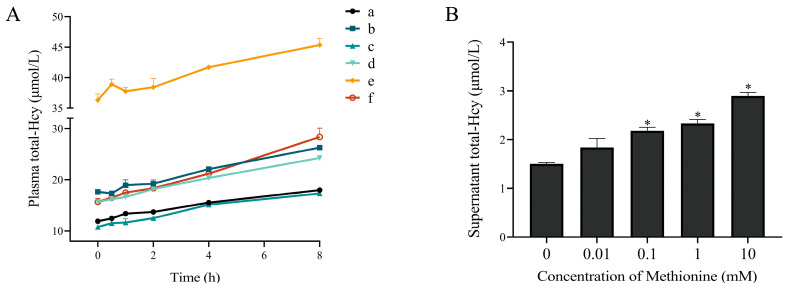
(**A**) The concentrations of plasma total-Hcy after whole blood kept at room temperature for different durations. Plasma total-Hcy content of each whole blood sample (a–f) increased with increasing storage time (0~8 h) at room temperature. (**B**) Effect of methionine on total-Hcy production from erythrocytes. Addition of 0.01~10 mM methionine led to a concentration-dependent increase of total-Hcy in the supernatant. * *p* < 0.05 vs. 0 mM methionine group.

The initial level of plasma total-Hcy from six volunteers ranged from 10.8 to 36.3 μM. The plasma total-Hcy content of each whole blood sample increased with increasing storage time at room temperature (Figure 1A). Linear regression analysis showed that the plasma total-Hcy concentrations increased linearly with time (Supplementary Table S1).

### 3.2. The Production of Homocysteine from Erythrocytes and Leukocytes

Erythrocytes and leukocytes were isolated from whole blood samples and incubated in serum-free medium for 0 h or 3 h, respectively. The results showed that total-Hcy released from erythrocytes was 104 ± 1.95 nmol/10^6^ cells and 670 ± 14.8 nmol/10^6^ cells at 0 h and 3 h time points, respectively, while total-Hcy released from leukocytes was 137 ± 19.7 nmol/10^6^ cells and 171 ± 7.61 nmol/10^6^ cells at 0 h and 3 h time points, respectively. 

The freshly isolated erythrocytes were suspended in plasma and incubated at room temperature for 3 h. The results showed that the concentration of plasma total-Hcy was 21.7 ± 0.30 nmol/mL, in which the r-Hcy level was only 0.05 ± 0.01 nmol/mL. The level of o-Hcy was then calculated to be 21.65 nmol/mL. The intracellular concentration of total-Hcy was 0.59 ± 0.02 nmol/mL, in which the r-Hcy was 0.59 ± 0.03 nmol/mL.

### 3.3. Incubation of Erythrocytes with Methionine Increased Total-Hcy Production

Incubation of erythrocytes with different concentrations of methionine (0.1, 1, 10 mM) led to a significant increase of total-Hcy in the supernatant (*p* < 0.05) (Figure 1B).

### 3.4. r-Hcy and Methionine Inhibited the Production of Erythrocytes ROS Induced by Rosup

**Figure 2:**
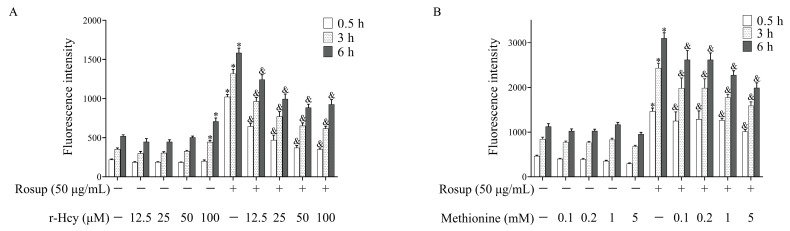
The effects of r-Hcy and methionine on ROS production in Rosup-treated erythrocytes. Incubation of Rosup-treated erythrocytes with 12.5~100 μM r-Hcy (**A**) and 0.1~5 mM methionine (**B**) for 0.5~6 h could significantly decrease ROS level in a concentration-dependent manner (*n* = 6). * *p* < 0.05 vs. control group (the group without Rosup, r-Hcy, or methionine treatment), ^&^ *p* < 0.05 vs. Rosup group (Rosup treatment alone).

The results showed that ROS level in Rosup-treated erythrocytes was significantly higher than that of the control for various durations of time (0.5 h, 3 h, 6 h) (Figure 2A,B). Incubation of Rosup-treated erythrocytes with 12.5~100 μM r-Hcy resulted in a concentration-dependent decrease of ROS production (*p* < 0.05) (Figure 2A). The 0.1~5 mM methionine treatment also significantly inhibited erythrocytes ROS production induced by Rosup (*p* < 0.05) (Figure 2B). Additionally, the 100 μM r-Hcy treatment could increase erythrocyte ROS production after 3 or 6 h incubation, compared with control (*p* < 0.05) (Figure 2A). The time-dependent increases in ROS production were also shown, not only in treatment groups, but also in the control group (*p* < 0.05).

### 3.5. The Effects of r-Hcy and Methionine on Erythrocytes Osmotic Fragility under Oxidative Stress

**Figure 3:**
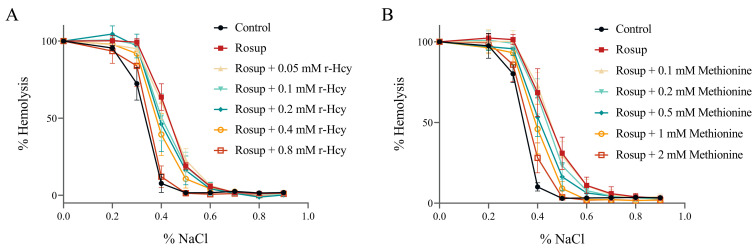
The protective effects of r-Hcy and methionine on erythrocytes osmotic fragility under oxidative stress. (**A**) The fragility curve shifted to the right after treatment of erythrocytes with Rosup for 6 h, with 50% hemolysis at 0.43 ± 0.01% NaCl solution. The 0.05~0.8 mM r-Hcy treatment resulted in the fragility curves of Rosup-treated erythrocytes shifted back to the left. (**B**) The fragility curve shifted to the right after treatment of erythrocytes with Rosup for 6 h, with 50% hemolysis at 0.45 ± 0.03% NaCl solution, and 0.1~2 mM methionine treatment inhibited Rosup-induced erythrocytes osmotic fragility elevation in a dose-dependent manner (*n* = 4).

The control erythrocytes showed 50% hemolysis at 0.33 ± 0.01% to 0.34 ± 0.01% NaCl solution. The fragility curve shifted to the right after treatment of erythrocytes with Rosup for 6 h, with 50% hemolysis at between 0.43 ± 0.01% and 0.45 ± 0.03% NaCl solution (Figure 3A,B). Methionine or r-Hcy treatment inhibited the Rosup-induced elevation of erythrocyte osmotic fragility (Figure 3A,B) (Supplementary Tables S2 and S3). The 50% hemolysis in Rosup + methionine (1mM) group, Rosup + methionine (2mM) group, and Rosup + r-Hcy (0.8 mM) were at 0.39 ± 0.03%, 0.36 ± 0.02%, and 0.34 ± 0.02% NaCl solution, respectively, which were significantly lower than those in the Rosup treatment group (*p* < 0.05) (Supplementary Tables S2 and S3).

### 3.6. The Effects of r-Hcy and Methionine on Ferryl Hb Decay

**Figure 4:**
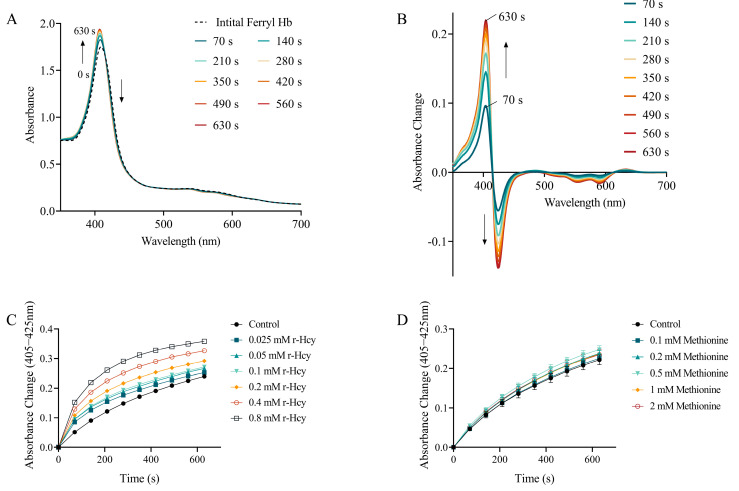
The effects of r-Hcy and methionine on ferryl Hb decay. (**A**) Representative absorbance spectra taken every 70 s after the addition of 0.8 mM r-Hcy (different color line represented different time point). (**B**) Absorbance change curves derived from original absorbance spectra in which initial ferryl Hb (Fe^4+^) spectra were set to zero. Time courses of ferryl (Fe^4+^) reduction at different concentrations of r-Hcy (**C**) or methionine (**D**). Addition of 0.025~0.8 mM r-Hcy to ferryl Hb (Fe^4+^) solution led to conversion of ferryl Hb (Fe^4+^) to ferric Hb (Fe^3+^) in concentration-dependent and time-dependent manners. However, the addition of 0.1~2 mM methionine to ferryl Hb (Fe^4+^) solution did not significantly change the heme state, compared with control (*p* > 0.05), except that 0.5 mM methionine caused a slight but significantly increased conversion of ferryl Hb (Fe^4+^) to ferric Hb (Fe^3+^). The detailed data can be found in Supplementary Tables S4 and S5.

Here, 405 nm and 425 nm were assigned to the Soret bands of ferric Hb (Fe^3+^) and ferryl Hb (Fe^4+^), respectively. An increase at 405 nm with a decrease at 425 nm indicates the typical shifting with the reduction of ferryl Hb (Fe^4+^) to ferric Hb (Fe^3+^) (Figure 4A,B). The addition of different concentrations of r-Hcy to ferryl Hb solution led to conversion of ferryl Hb (Fe^4+^) to ferric Hb (Fe^3+^) in concentration-dependent and time-dependent manners (*p* < 0.05) (Figure 4C) (Supplementary Table S4). However, the addition of 0.1~2 mM methionine to ferryl Hb (Fe^4+^) solution did not significantly change the heme state, compared with control (*p* > 0.05) (Figure 4D), except that 0.5 mM methionine caused a slight but significant increased conversion of ferryl Hb (Fe^4+^) to ferric Hb (Fe^3+^) (Figure 4D) (Supplementary Table S5).

### 3.7. The Effects of r-Hcy and Methionine on MetHb Formation in Rosup-Treated Erythrocytes

**Figure 5:**
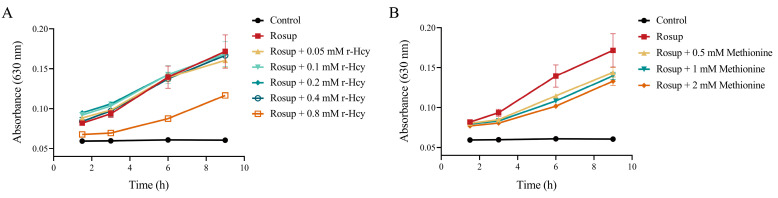
Effects of r-Hcy and methionine on MetHb formation in Rosup-treated erythrocytes (*n* = 3). The absorbance at 630 nm represented the content of MetHb. (**A**) r-Hcy (0.8 mM) treatment for 3~9 h significantly inhibited MetHb formation induced by Rosup. (**B**) Treatment with 0.5~2 mM methionine for 6~9 h significantly decreased formation of MetHb in Rosup-treated erythrocytes.

Incubation of erythrocytes with 0.05~0.8 mM r-Hcy or 0.5~2 mM methionine for 1.5~9 h did not influence MetHb formation, compared with control group (*p* > 0.05) (Supplementary Figure S1). Rosup treatment significantly increased MetHb formation in erythrocytes at all time intervals (*p* < 0.05) (Figure 5) (Supplementary Table S6). Co-incubation of erythrocytes with Rosup and 0.8 mM r-Hcy for 3~9 h significantly decreased MetHb level, compared with Rosup treatment alone (*p* < 0.05) (Figure 5A). Co-incubation of erythrocytes with Rosup and 0.5~2 mM methionine for 6~9 h significantly reduced MetHb formation, compared with Rosup treatment alone (*p* < 0.05) (Figure 5B).

### 3.8. The Alterations of Total-Hcy and GSH Levels in Erythrocytes after Treatment with Rosup

**Figure 6:**
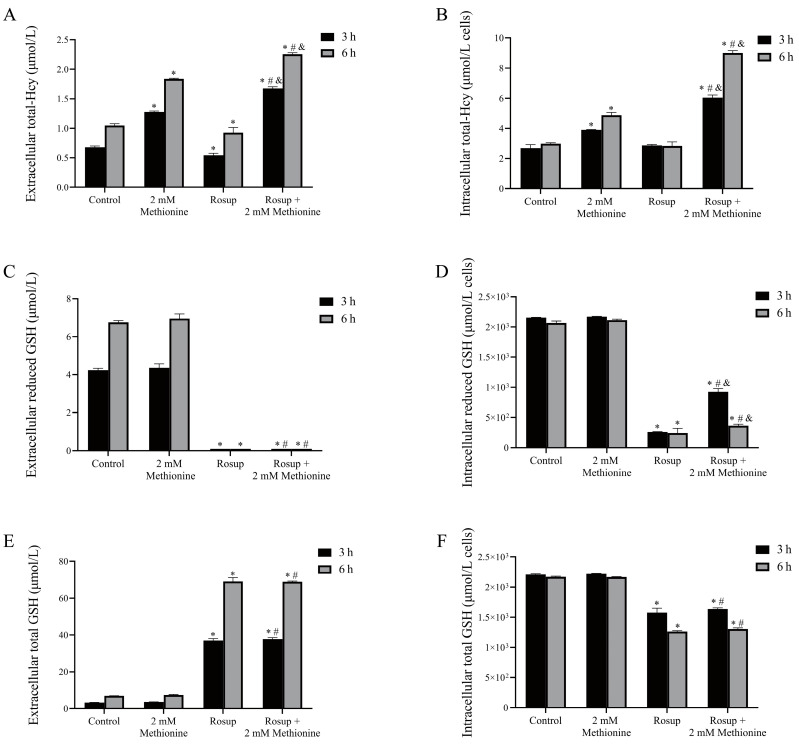
The alterations of total-Hcy and GSH levels in erythrocytes after treatment with Rosup in the presence or absence of methionine (*n* = 3). (**A**) The concentration of extracellular total-Hcy reduced slightly after Rosup treatment, compared with that of control. Methionine (2 mM) plus Rosup treatment for 3 h or 6 h resulted in a significant increase of extracellular total-Hcy contents, compared with 2 mM methionine or Rosup treatment alone (*p* < 0.05). (**B**) Intracellular total-Hcy level did not alter significantly after Rosup treatment (*p* > 0.05). Methionine (2 mM) plus Rosup treatment for 3 h or 6 h caused a significant increase of intracellular total-Hcy contents, compared with 2 mM methionine or Rosup treatment alone (*p* < 0.05). (**C**–**F**) Rosup treatment significantly decreased intracellular and extracellular reduced GSH levels compared with control (*p* < 0.05). Methionine treatment alone did not significantly change the intracellular and extracellular reduced GSH and total GSH levels (*p* > 0.05) but significantly inhibited the decrease of intracellular reduced GSH levels induced by Rosup. * *p* < 0.05 vs. control group, ^# ^*p* < 0.05 vs. 2 mM Methionine group, ^&^ *p* < 0.05 vs. Rosup group.

The concentration of extracellular total-Hcy decreased slightly after Rosup treatment, compared with that of control (Figure 6A). At the 3 h time point, the concentrations of extracellular total-Hcy were 0.68 ± 0.02 μmol/L and 0.54 ± 0.03 μmol/L in the control and Rosup treatment group. At the 6 h time point, the concentrations of extracellular total-Hcy were 1.05 ± 0.03 μmol/L and 0.93 ± 0.09 μmol/L in the control and Rosup treatment group, respectively. Intracellular total-Hcy level did not alter significantly after Rosup treatment (*p* > 0.05) (Figure 6B). Methionine (2 mM) plus Rosup treatment for 3 h or 6 h resulted in a significant increase of extracellular and intracellular total-Hcy contents, compared with 2 mM methionine or Rosup treatment alone (*p* < 0.05) (Figure 6A,B).

Rosup treatment significantly decreased intracellular and extracellular reduced GSH levels, compared with control (*p* < 0.05) (Figure 6C,D). Methionine treatment alone did not significantly change the intracellular and extracellular reduced GSH and total GSH levels (*p* > 0.05) (Figure 6C–F) but could significantly inhibit the decrease of intracellular reduced GSH levels induced by Rosup (*p* < 0.05) (Figure 6C,D).

In addition, the concentrations of extracellular total-Hcy were significantly higher after the 6 h incubation than those after 3 h incubation in all treatment and control groups (*p* < 0.05) (Figure 6A). Extracellular reduced GSH levels and total GSH levels were also higher after the 6 h incubation than after 3 h incubation in control samples (*p* < 0.05) (Figure 6C,E).

## 4. Discussion

In the present work, the time-dependent increases in plasma total-Hcy contents were observed after whole blood was kept at room temperature, which was consistent with previously reported [11]. To further elucidate the origin of plasma total-Hcy, erythrocytes and leukocytes were separated and incubated, and the results showed that after a three- hour incubation, total-Hcy released from erythrocytes increased by about 6 times compared to the initial level, while total-Hcy released from leukocytes was only about 20% higher than the initial level. Considering that the amount of erythrocyte in the peripheral blood are about 1000 times more than that of leukocytes, the total-Hcy released from erythrocytes was estimated to be thousands of times more than that released from leukocytes. This result indicated that erythrocytes were the main type of blood cells that produced homocysteine and responsible for the increase of plasma total-Hcy level after whole blood was kept at room temperature for an extended time. 

The function of mature erythrocytes is the transport of oxygen from the lungs to the tissues, providing cells with oxygen, which continuously exposes them to both endogenous and exogenous sources of ROS during their lifespan. To minimize the damaging effect of ROS, erythrocytes have an extensive antioxidant system involving both antioxidants such as GSH and ascorbic acid, and enzymes including superoxide dismutase, catalase, glutathione peroxidase, and peroxiredoxin-2 [31]. Among them, GSH, as the main form of intracellular functional sulfhydryl group (SH) carrier, can form a disulfide bond (SS) after being oxidized by ROS [32]. This disulfide bond (SS) can then be reduced back to the free sulfhydryl group (SH) by the NADPH-reducing system. By this means, sulfhydryl/disulfide (SH/SS) homeostasis is maintained, which protects the erythrocyte from oxidative damaging [33]. Similar to GSH, homocysteine contains a free sulfhydryl group (SH), making it very possible to contribute to maintaining the thiol redox balance in erythrocytes.

Rosup was widely used to induce oxidative stress in cell studies [22,23]. It was employed in this study to clarify whether r-Hcy would act as an antioxidant in the erythrocyte. The results showed that r-Hcy could significantly decrease Rosup-induced ROS production, indicating that r-Hcy might serve as an ROS scavenger when the erythrocytes experienced excess ROS attack. Meanwhile, incubation of erythrocytes with r-Hcy at a higher level (100 μM) induced an observable increase in ROS production without the presence of Rosup. This result suggested that the prooxidative or antioxidative effects of r-Hcy might depend on its concentration. It is well accepted that oxidative stress is produced when the equilibrium between ROS and antioxidants is broken with the state in favor of the former. Meanwhile, antioxidants may behave as a free radical producer if concentration is high [34,35]. Therefore, when the erythrocytes were in redox equilibrium without excess oxidative stress, adding too much r-Hcy might induce the production of ROS through autooxidation of r-Hcy by transferring an electron to an oxygen molecule. A recent in-test-tube study reported that metal-induced oxidation of r-Hcy (1~5 mM) forming disulfides in the presence of horseradish peroxidase resulted in the generation of ROS [36]. These results show that the supplement of reducing agents is not necessarily beneficial when the cells are in the redox equilibrium. 

Methionine is the sole precursor for homocysteine in humans [37,38]. Our findings showed that incubating erythrocytes with methionine resulted in a steady release of total-Hcy in a methionine concentration-dependent manner, indicating that homocysteine could be derived from methionine through the transmethylation pathway in erythrocytes. The observed steady release of total-Hcy in blood (Supplementary Table S1) also indicated that methionine was steadily metabolized, which would produce a stable antioxidative effect within erythrocytes without inducing redox disequilibrium. In fact, our present work demonstrated that methionine treatment indeed decreased ROS production in a dose-dependent manner in Rosup-treated erythrocytes and did not influence ROS production in control erythrocytes. In addition, the time-dependent increase of ROS production was shown not only in treatment groups but also in control groups, indicating that erythrocytes were actively metabolizing during the experiment. Auto-oxidation of Hb is considered to be the major source of superoxide anion production in erythrocytes and can be produced continuously [39]. With the elongation of incubation time, ROS was produced within erythrocytes and captured by DCFH-DA, which was indicated by increased fluorescence intensity. This result also indicated that the steady release of total-Hcy in blood might partially be due to the need for antioxidation in erythrocytes.

Erythrocyte osmotic fragility is an indirect method of assessing oxidative stress [24,25]. The present study showed that r-Hcy and methionine could inhibit Rosup-induced erythrocyte osmotic fragility increase in a dose-dependent manner, indicating that r-Hcy and its precursor methionine contribute to maintaining the erythrocyte deformability. This could also be explained by the potential protective effect on the Band 3 protein (B3p) since it contains an abundance of cysteine with the SH group. B3p is abundant in erythrocyte membranes and involved in maintaining the osmotic and normal deformability of erythrocytes [40,41].

Erythrocytes are highly enriched in Hb, which is a tetrameric protein consisting of two α and two β subunits (α2β2) [42]. Each subunit has a protein part, and an iron-containing protoporphyrin moiety called heme, and each ferrous ion (Fe^2+^) in the heme can bind to one oxygen molecule. Ferrous (Fe^2+^) or ferric (Fe^3+^) Hb can be oxidized by H_2_O_2_ to form ferryl heme iron (Fe^4+^) [43,44,45]. The highly reducing environment within erythrocytes prevents the oxidation of Hb and maintains the functional ferrous (Fe^2+^) form. In the present study, we further investigate the potential role of r-Hcy in H_2_O_2_-induced Hb oxidation and decay by ferryl Hb decay assay that was performed in test-tube. The results showed that r-Hcy treatment could enhance the reduction of Hb from the ferryl (Fe^4+^) form to the ferric (Fe^3+^) form, suggesting that r-Hcy might play a role in restoring the redox state of Hb directly. However, methionine treatment did not influence ferryl Hb decay directly. This in-test-tube experiment demonstrated that it was r-Hcy, but not methionine, which increased the reduction rate of ferryl Hb (Fe^4+^) since the methionine metabolic enzyme system was absent in test-tube. Therefore, the antioxidative effects of methionine in erythrocytes could be attributed to its metabolite r-Hcy. To confirm the antioxidative effects of r-Hcy and methionine on intracellular Hb oxidation, the experiment of MetHb formation in Rosup-treated erythrocytes was carried out as well. r-Hcy (800 μM) or methionine (0.5~2 mM) treatment could significantly inhibit MetHb formation in erythrocytes induced by Rosup. This result further confirmed that the antioxidative effect of methionine was attributed to its metabolite r-Hcy in erythrocytes.

It is known that oxygen has an inherent ability to oxidize the ferrous heme iron (Fe^2+^), and the oxidation rate of ferrous Hb (Fe^2+^) to ferric (Fe^3+^) Hb may increase with the increasing partial pressure of oxygen, superoxide is produced at the same time [46]. Therefore, if the superoxide was not removed immediately, it would be detrimental to erythrocytes. Superoxide, H_2_O_2_, hydroxyl free radicals, ferryl Hb (Fe^4+^), etc., generated by these redox reactions can damage erythrocyte membrane proteins, lipids, and the cytoskeleton, leading to erythrocyte shape and deformability impairment. At the same time, specific modifications in human Hb, such as oxidation of the sulfhydryl group (SH) of βCys-93 and βCys-112, have been observed under oxidative stress [47]. Therefore, it is reasonable to suggest that the continuous production of r-Hcy might serve as a supplementary intracellular antioxidant besides GSH. The present work showed that Rosup treatment significantly decreased intracellular reduced GSH and total GSH, but significantly increased total extracellular GSH, indicating that oxidative stress might exhaust intracellular reduced GSH and cause GSH leakage from erythrocytes, resulting in the reducing capacity impairment in erythrocytes. Treatment with methionine significantly restored intracellular reduced GSH level. Since the addition of methionine could significantly increase the total-Hcy level in Rosup-treated erythrocytes, we supposed that the transmethylation pathway of methionine might be activated under oxidative stress and play a supplementary role in the antioxidative system of the erythrocyte. The demethylation of methionine to homocysteine actually supplies a more reductive sulfhydryl group (SH). Our present work also showed that intracellular homocysteine was in reduced form (r-Hcy, about 100%), but extracellular homocysteine was mainly in oxidized form (o-Hcy, about 99.8%). In addition, the content of total-Hcy in the culture supernatant was 37-fold higher than in erythrocytes after incubation for 3 h. Therefore, r-Hcy is produced from methionine within erythrocytes, then oxidized to o-Hcy within cells, and subsequently released into the extracellular fluid. This might be the mechanism of how methionine and homocysteine play a role in maintaining intracellular redox status. 

**Figure 7:**
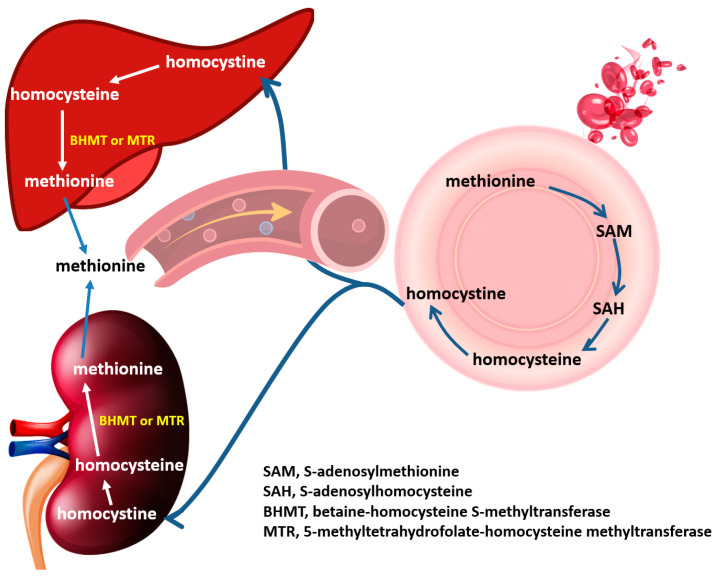
Diagram of erythrocytic-renal cycle and erythrocytic-hepatic cycle. In erythrocytes, methionine is converted to SAM, which is a methyl donor for numerous reactions. After losing its methyl group, SAM becomes SAH, which is then converted to homocysteine. Homocysteine exerts its antioxidative effect and converts to homocystine and then releases to extracellular fluid and transport in circulation to the kidney or liver, which shows enriched expression of BHMT and MTR. In the kidney or liver, homocysteine can be converted back to methionine by the addition of a methyl group. Finally, methionine is synthesized and returned to circulation for further utilization.

In the body, homocysteine is mainly metabolized back to methionine via a remethylation pathway in the presence of metabolic enzymes 5-methyltetrahydrofolate-homocysteine methyltransferase (MTR) or betaine--homocysteine S-methyltransferase (BHMT) [48]. A recent transcriptomics and antibody-based proteomics study demonstrated that BHMT was a biased expression in the kidney and liver, and MTR was ubiquitously expressed in the kidney, liver, and 25 other tissues [49,50]. Therefore, we proposed that homocystine (the main o-Hcy) released from erythrocytes would be transported by circulation to remote organs, such as the kidney and liver. Then, homocystine is reduced and remethylated to methionine catalyzed by BHMT and MTR within renal cells and hepatocytes. Thus, the methionine cycle is complete, and the body can make the best use of methionine, which can be called the erythrocytic-renal cycle or erythrocytic-hepatic cycle (Figure 7).

## 5. Conclusions

In conclusion, our present study demonstrates for the first time that erythrocytes metabolize methionine to homocysteine, which is responsible for the increase of plasma total-Hcy level after whole blood is kept at room temperature for an extended time. Homocysteine produced in erythrocytes contributes to intracellular antioxidative capability under oxidative stress, which is essential to prevent the oxidation of Hb and maintain the erythrocyte shape and deformability. This research suggests that methionine may play antioxidative roles within erythrocytes, especially in those who suffer from glucose-6-phosphate dehydrogenase (G6PD) deficiency, which renders erythrocytes susceptible to oxidative stress. Further research should be considered on how methionine demethylation is regulated by redox status within erythrocytes.

## Data Availability

Data is contained within the article or Supplementary Material.

## Supplementary Materials

The following supporting information can be downloaded at: https://www.mdpi.com/article/10.3390/antiox12010202/s1, Figure S1: The effects of r-Hcy and methionine on MetHb formation in control erythrocytes; Table S1: The release velocity of homocysteine in each whole blood sample from six volunteers during storge at room temperature; Table S2: The concentrations of NaCl solution leading to 50% hemolysis after treatment with r-Hcy (mean ± SD, *n* = 4); Table S3: The concentrations of NaCl solution leading to 50% hemolysis after treatment with methionine (mean ± SD, *n* = 4); Table S4: The effects of r-Hcy on ferryl Hb decay (absorbance change at 405–425 nm, mean ± SD, *n* = 3); Table S5: The effects of methionine on ferryl Hb decay (absorbance change at 405–425 nm, mean ± SD, *n* = 3); Table S6: The effects of r-Hcy and methionine on MetHb formation in Rosup-treated erythrocytes (represented by the absorbance at 630 nm, mean ± SD, *n* = 3).
