# Supplementary material for: Potential Antioxidative Activity of Homocysteine in Erythrocytes under Oxidative Stress
Source: Antioxidants (Basel). 2023 Jan 15;12(1):202. doi: 10.3390/antiox12010202 (PMC9855177; doi:10.3390/antiox12010202)
Supplement: Supplementary file 1 [file antioxidants-12-00202-s001.zip › antioxidants-2102829-supplementary.pdf]

Supplementary File

Table S1: The release velocity of homocysteine in each whole blood sample from six volunteers during storge at room temperature.

Volunteers	Equation	R square	Release velocity (μM/h)
a	Y = 0.7097 × X + 0.5153	0.9818	1.225
b	Y = 1.143 × X − 0.4263	0.9654	0.717
c	Y = 0.8130 × X + 0.3158	0.9518	1.129
d	Y = 1.077 × X + 0.1115	0.9891	1.189
e	Y = 0.9964 × X + 1.010	0.8889	2.006
f	Y = 1.564 × X − 0.1739	0.9687	1.390

Table S2: The concentrations of NaCl solution leading to 50% hemolysis after treatment with r-Hcy （mean ± SD, n=4）.

Groups	% NaCl
Control	0.33 ± 0.01
Rosup	0.43 ± 0.01
Rosup + 0.05 mM r-Hcy	0.43 ± 0.02
Rosup + 0.1 mM r-Hcy	0.41 ± 0.03
Rosup + 0.2 mM r-Hcy	0.41 ± 0.02
Rosup + 0.4 mM r-Hcy	0.38 ± 0.03
Rosup + 0.8 mM r-Hcy	0.34 ± 0.02*

* $p < 0.05$ *vs* Rousp group.

Table S3: The concentrations of NaCl solution leading to 50% hemolysis after treatment with methionine （mean ± SD, n=4）.

Groups	% NaCl
Control	0.34 ± 0.01
Rosup	0.45 ± 0.03
Rosup + 0.1 mM Methionine	0.45 ± 0.02
Rosup + 0.2 mM Methionine	0.44 ± 0.02
Rosup + 0.5 mM Methionine	0.41 ± 0.03
Rosup + 1 mM Methionine	0.39 ± 0.03*
Rosup + 2 mM Methionine	0.36 ± 0.02*

* $p < 0.05$ *vs* Rosup group.

Table S4: The effects of r-Hcy on ferryl Hb decay (Absorbance change at 405-425nm, mean ± SD, n=3)

	Groups (r-Hcy, mM)						
Time (s)	Control	0.025	0.050	0.1	0.2	0.4	0.8
70	0.051 ± 0.003	0.085 ± 0.001*	0.095 ± 0.003*	0.096 ± 0.006*	0.108 ± 0.002*	0.130 ± 0.004*	0.152 ± 0.002*
140	0.090 ± 0.005	0.125 ± 0.001*	0.136 ± 0.003*	0.139 ± 0.006*	0.157 ± 0.003*	0.187 ± 0.005*	0.219 ± 0.002*
210	0.122 ± 0.005	0.154 ± 0.001*	0.165 ± 0.003*	0.170 ± 0.007*	0.191 ± 0.003*	0.224 ± 0.004*	0.261 ± 0.001*
280	0.148 ± 0.006	0.176 ± 0.001*	0.188 ± 0.004*	0.194 ± 0.007*	0.217 ± 0.002*	0.252 ± 0.005*	0.291 ± 0.003*
350	0.171 ± 0.006	0.195 ± 0.001*	0.207 ± 0.003*	0.213 ± 0.006*	0.236 ± 0.003*	0.273 ± 0.006*	0.312 ± 0.003*
420	0.191 ± 0.006	0.212 ± 0.001*	0.224 ± 0.003*	0.230 ± 0.006*	0.254 ± 0.002*	0.290 ± 0.005*	0.328 ± 0.003*

490	0.209 ± 0.006	0.227 ± 0.001*	0.240 ± 0.003*	0.245 ± 0.006*	0.268 ± 0.003*	0.305 ± 0.005*	0.340 ± 0.003*
560	0.225 ± 0.006	0.241 ± 0.000*	0.254 ± 0.004*	0.259 ± 0.007*	0.280 ± 0.001*	0.316 ± 0.006*	0.350 ± 0.003*
630	0.240 ± 0.007	0.253 ± 0.001*	0.266 ± 0.005*	0.271 ± 0.006*	0.292 ± 0.001*	0.326 ± 0.006*	0.358 ± 0.004*

* $p < 0.05$ *vs* Control group.

Table S5: The effects of methionine on ferryl Hb decay (Absorbance change at 405-425nm, mean ± SD, n=3)

	Groups (Methionine, mM)					
Time (s)	Control	0.1	0.2	0.5	1	2
70	0.046 ± 0.005	0.047 ± 0.002	0.050 ± 0.002	0.055 ± 0.003	0.050 ± 0.003	0.050 ± 0.003
140	0.083 ± 0.008	0.083 ± 0.003	0.088 ± 0.007	0.096 ± 0.005	0.089 ± 0.003	0.090 ± 0.008
210	0.112 ± 0.009	0.113 ± 0.003	0.120 ± 0.007	0.128 ± 0.006	0.120 ± 0.003	0.122 ± 0.010
280	0.136 ± 0.010	0.138 ± 0.002	0.146 ± 0.008	0.155 ± 0.008*	0.146 ± 0.004	0.148 ± 0.011
350	0.157 ± 0.010	0.159 ± 0.003	0.170 ± 0.007	0.179 ± 0.008*	0.168 ± 0.005	0.170 ± 0.012
420	0.176 ± 0.011	0.178 ± 0.002	0.190 ± 0.008	0.200 ± 0.009*	0.189 ± 0.004	0.190 ± 0.012
490	0.193 ± 0.012	0.196 ± 0.002	0.208 ± 0.007	0.219 ± 0.010*	0.207 ± 0.003	0.207 ± 0.012
560	0.208 ± 0.011	0.211 ± 0.001	0.224 ± 0.007	0.235 ± 0.011*	0.222 ± 0.002	0.222 ± 0.013
630	0.222 ± 0.012	0.225 ± 0.001	0.238 ± 0.007	0.248 ± 0.010*	0.237 ± 0.001	0.235 ± 0.013

* $p < 0.05$ *vs* Control group.

Table S6: The effects of r-Hcy and methionine on MetHb formation in Rosup-treated erythrocytes (represented by the absorbance at 630 nm, mean ± SD, n=3)

	Time (h)			
Groups	1.5	3	6	9
Control	0.059 ± 0.001	0.060 ±0.000	0.061 ± 0.001	0.060 ± 0.002
Rosup	0.082 ± 0.001*	0.093 ± 0.004*	0.139 ± 0.014*	0.172 ± 0.021*
Rosup + 0.05 mM r-Hcy	0.088 ± 0.003	0.098 ± 0.003	0.139 ± 0.005	0.160 ± 0.009
Rosup + 0.1 mM r-Hcy	0.092 ± 0.004	0.103 ± 0.005	0.143 ± 0.011	0.168 ± 0.016
Rosup + 0.2 mM r-Hcy	0.095 ± 0.002	0.106 ± 0.001	0.142 ± 0.001	0.168 ± 0.002
Rosup + 0.4 mM r-Hcy	0.084 ± 0.005	0.097 ± 0.008	0.137 ± 0.004	0.167 ± 0.002
Rosup + 0.8 mM r-Hcy	0.068 ± 0.001	0.069 ± 0.000#	0.087 ± 0.003#	0.116 ± 0.002#
Rosup + 0.5 mM Methionine	0.080 ± 0.001	0.085 ± 0.000	0.115 ± 0.003#	0.144 ± 0.006#
Rosup + 1 mM Methionine	0.079 ± 0.001	0.083 ± 0.003	0.108 ± 0.003#	0.140 ± 0.005#
Rosup + 2 mM Methionine	0.077 ± 0.001	0.080 ± 0.001	0.102 ± 0.001#	0.133 ± 0.005#

* $p < 0.05$ *vs* Control group, # $p < 0.05$ *vs* Rosup group.

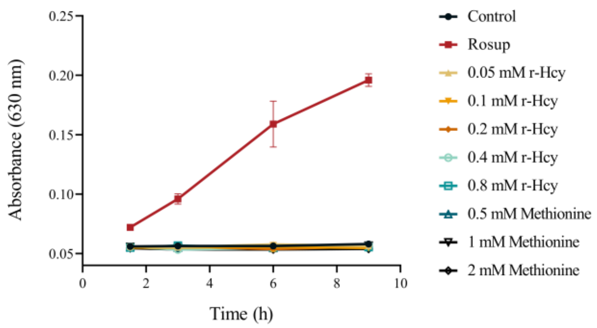

Figure S1: The effects of r-Hcy and methionine on MetHb formation in control erythrocytes. After treatment with different concentrations of methionine (0.5, 1, 2mM) or r-Hcy (0.05, 0.1, 0.2, 0.4, 0.8 mM) for 1.5 h, 3 h, 6 h and 9 h, the absorbance at 630 nm did not change significantly compared with Control ($p > 0.05$).
